# Three-dimensional speckle-tracking imaging for the prognosis of childhood-onset systemic lupus erythematosus: a pilot study

**DOI:** 10.3389/fped.2025.1510852

**Published:** 2025-04-09

**Authors:** Xiaoyuan Feng, Ping Zhou, Yan Ding, Jing Peng, Tao Wang

**Affiliations:** ^1^ Department of Echocardiography, Wuhan Children’s Hospital, Tongji Medical College, Huazhong University of Science & Technology, Wuhan, China; ^2^ Department of Children’s Critical Care Medical Center, Wuhan Children’s Hospital, Tongji Medical College, Huazhong University of Science & Technology , Wuhan, China; ^3^ Department of Rheumatology, Wuhan Children’s Hospital, Tongji Medical College, Huazhong University of Science & Technology, Wuhan, China; ^4^ Department of Cardiothoracic Surgery, Wuhan Children’s Hospital, Tongji Medical College, Huazhong University of Science & Technology, Wuhan, China

**Keywords:** childhood-onset systemic lupus erythematosus, cardiac damage, 3D speckle tracking imaging, myocardial comprehensive index, prognosis

## Abstract

**Background:**

To assess the early alterations in the architecture and performance of the left ventricle for childhood-onset systemic lupus erythematosus (cSLE) patients utilizing three-dimensional speckle tracking imaging (3D-STI).

**Methods:**

The aggregate of 31 cSLE patients were recruited and categorized into two groups based on the SLE disease activity index (SLEDAI) score: the mild-to-moderate group (≤12, *n* = 14) and the severe group (>12, *n* = 17). Univariate as well as multivariate logistic regression were used to investigate the relationship between 3D-STI parameters and the activity of the disease. Four diagnostic patterns were employed to amalgamate 3D-STI data (global longitudinal strain, GLS, and left ventricular twist angle, LVtw): isolation, series, parallel, and integration, subsequently leading to the development of a 3D myocardial comprehensive index (3D-MCI). The primary aim was severe disease activity, whereas the secondary objectives were growth failure, lupus nephritis, hypocomplementemia, and serious hematological issues.

**Results:**

In the multivariate analysis, GLS and LVtw emerged as significant indicators of severe disease activity (*p* = 0.028 and *p* = 0.047). The comprehensive method, which integrates GLS with LVtw value using the logistic algorithm, achieves a balanced sensitivity and specificity of 81.4% and 94.1%, respectively. Subsequently, the 3D-MCI is computed as follows: 7.650–0.367*GLS (%) - 0.281*LVtw (°). Furthermore, the 3D-MCI exhibited a strong significant correlation with both the primary endpoint and the secondary outcomes.

**Conclusions:**

3D-STI technology may facilitate the early detection of cardiac injury in individuals with cSLE, whereas 3D-MCI serves as suitable prognostic indicators for cSLE patients.

## Introduction

Childhood-onset systemic lupus erythematosus (cSLE) is an uncommon, persistent, and devastating systemically immunological disorder (1, 2). In comparison to individuals with adult-onset illness, those with cSLE have elevated disease activity, a greater prevalence of renal and neuropsychiatric commitment, and experience damage progression more swiftly (3, 4). Cardiovascular impairment may be a primary contributor to the rising death rates observed annually in people with cSLE and adult SLE. Furthermore, prior researches had shown that the cardiovascular system is significantly impacted during the first stages of the disease, and any intervention aimed at reducing activity may alleviate the cardiovascular burden and thus decrease death rates (5, 6). Nevertheless, early detection of cardiovascular disease may still be hindered by insufficient equipment.

Echocardiography is extensively utilized for assessing cardiac structure as well as function because to its distinctive benefits, including its flexibility and minimally invasive characteristics; yet, it is unable to identify early-stage alterations in cardiovascular damage throughout illness progression (7, 8). Three-dimensional speckle tracking imaging (3D-STI), a novel technology that surpasses traditional echocardiography, may assess cardiovascular load for many illnesses at an earlier stage (9, 10). Additionally, a prior study effectively utilized 3D-STI to assess early alterations in left ventricular performance for adult SLE patients (11). However, there is a lack of knowledge about the application of 3D-STI for cardiovascular impairment in cSLE patients. Consequently, this study employed 3D-STI to assess the impact of SLE on the cardiovascular system, investigate its application worth, and provide an objective diagnostic foundation for clinical utilization.

## Materials and methods

### Participants

This research included 31 individuals with cSLE who were treated from May 2023 to July 2024. All patients fulfilled the diagnostic criteria for SLE set out by the American College of Rheumatology (ACR) in 1997. The criteria for inclusion were: (1) aligned with the SLE classification criteria established by the ACR; (2) patients diagnosed with SLE were under 18 years diagnostically and remain under 18 years of age currently; (3) patients in the SLE cohort received consistent treatment and exhibited relative stability in their condition; (4) all patients and their families were duly informed, and written informed consent was secured. Exclusion criteria: (1) concurrent hypertension, diabetes mellitus, heart illness, or renal disease; (2) other autoimmune disorders; (3) individuals with inadequate acoustic windows. The Ethics Committee of Wuhan Children's Hospital evaluated and approved this work.

Medical data, encompassing age at diagnosis, illness duration, corticosteroid and hydroxychloroquine usage, and laboratory results, were assessed. Alongside demographic data, SLE disease activity was evaluated utilizing the SLE Disease Activity Index (SLEDAI), categorizing all patients into two groups based on the SLEDAI score: mild-to-moderate group (SLEDAI <12 points, *n* = 14) and severe group (SLEDAI >12 points, *n* = 17). The categorization of nutritional status was determined using body mass index for age *z*-scores and height for age *z*-scores, employing the World Health Organization Anthro Plus 3.2.2 program.

### Echocardiography

All subjects had echocardiographic evaluation conducted by individuals with over 10 years of expertise. Color Doppler ultrasonography (GE Vivid E9, probe: 4 V, frequency: 1.5–4.0 MHz) was employed, and the integrated 3D-STI analysis program was utilized for post-processing. Following the patient's instruction to continue holding their breath for the visualization of a two-dimensional picture (clear apical four-chambers), the 4D mode was activated, and three ongoing, reliable, full cardiac rhythms were rapidly and seamlessly acquired. The frame rate was subsequently calibrated to 40% of the participant's maximum heart rate to ensure accurate speckle detection. In the 4D auto LVQ (LV volume quantification) mode, the software autonomously delineated the borders of the left ventricular wall (endocardium and epicardium) during the whole cardiac cycle, with manual adjustments made as needed. The subsequent 3D data were acquired: left ventricular end-diastolic volume (LVEDV), left ventricular end-systolic volume (LVESV), left ventricular ejection fraction (LVEF), spherical index (SPI), and left ventricular end-diastolic mass (LV EDmass). Additionally, the subsequent 3D strain characteristics were concurrently acquired: global longitudinal strain (GLS), global circumferential strain (GCS), global area strain (GAS), global radial strain (GRS), left ventricular twist angle (LVtw), torsion (Tor), and peak strain dispersion (PSD). Additionally, GLS, GAS, GRS, and GCS were computed as the mean of the regional values from the 17 myocardial segments, and a color-coded 17-segment bull's-eye plot was generated.

### Diagnosis strategy

The present study created four separate diagnostic strategies: (I) employing the GLS value and LVtw independently; (II) combining them in series; (III) combining them in parallel; and (IV) integrating them. As the isolation technique, the GLS and the LVtw were employed as indicator for severe SLE activity, respectively. The serial technique functioned by recognizing patients with extreme activity just when both the GLS and the LVtw signified the existence. The parallel method identified individuals with significant activity based on either the GLS value or the LVtw indicating this diagnosis. The integration technique employed a logistic algorithm to amalgamate the GLS and the LVtw, resulting in a cohesive approach. The diagnostic results were subsequently established using a predetermined cutoff value, facilitating a dependable and precise evaluation.

### Outcomes

The primary endpoint of this study was the severe activity of SLE, as determined by the SELDAI score. The secondary results encompassed growth failure, lupus nephritis, hypocomplementemia, and serious hematological issues.

Growth failure was characterized by a height below −2 standard deviations according to the 2009 standards for Chinese children (12). The diagnosis of lupus nephritis follows the guidelines established by the Chinese Society of Pediatrics in 2010 (13). Additionally, low serum C3 levels (<0.80 g/L) and/or C4 levels (<0.10 g/L) were classified as hypocomplementemia. Severe hematological problems were characterized as macrophage activation syndrome, severe cytopenia (hemoglobin <80 g/L and/or platelets <50 × 10^9^/L), hemorrhage, or malignancy.

### Statistical analysis

All data analysis were performed using R statistical program. Continuous variables were presented as mean ± standard deviation or median (interquartile range), and comparisons were performed using either the student's *t*-test or the Mann–Whitney *U*-test. Categorical variables were expressed as frequencies and analyzed using Pearson's Chi-squared test. Logistic regression analysis was employed to identify the relationship between 3D-STI features and SLE activity. A study was performed to examine the correlation between diagnostic outcomes and the SLEDAI findings utilizing Pearson's Chi-squared test. The degree of agreement between them was assessed using Cohen's Kappa test. The receiver operating characteristic (ROC) curve was generated, and the area under the curve (AUC) was computed to assess the efficacy of the diagnostic patterns. The Delong test and McNemar test were employed to compare the AUCs, sensitivities, and specificities of diagnostic methods. Statistical significance was established as a two-sided *P*-value less than 0.05.

## Results

### Baseline characteristics

The baseline characteristics of the research population are presented in Table 1. In comparison to the mild-to-moderate group, patients in the severe group had a greater prevalence of positive results for dsDNA, nucleosome, and antiphospholipid, alongside reduced levels of C3 and C4. However, there were no difference in demographic data, nutritional status, utilization of corticosteroids as well as hydroxychloroquine.

**Table 1:** Baseline characteristics of the study population.

Characteristics	Severe group (*n* = 14)	Mild-to-moderate group (*n* = 17)	*P*-value
Age, years	12.4 ± 3.5	13.2 ± 2.8	0.472
Age of onset, years	9.9 ± 3.4	10.1 ± 1.9	0.796
Sex, female, *n* (%)	11 (78.6)	16 (94.1)	0.199
ZBA	0.8 ± 1.4	1.0 ± 1.3	0.668
Excess weight, *n* (%)	3 (21.4)	3 (17.6)	0.887
ZHA	1.5 ± 2.2	1.7 ± 2.0	0.785
Short stature, *n* (%)	1 (7.1)	1 (5.9)	0.791
SBP, mmHg	104.9 ± 14.4	104.3 ± 14.7	0.905
DBP, mmHg	67.8 ± 13.4	67.1 ± 11.6	0.873
Corticosteroid, *n* (%)	14 (100.0)	16 (94.1)	0.356
Dose, mg/kg	0.28 (0.15, 0.41)	0.23 (0.12, 0.49)	0.543
>0.5 mg/kg, *n* (%)	3 (21.4)	4 (23.5)	0.818
Hydroxychloroquine, *n* (%)	11 (78.6)	14 (82.4)	0.791
Dose, mg	200.0 (130.0, 250.0)	150.0 (137.5, 200.0)	0.349
Anti-dsDNA +, *n* (%)	11 (78.6)	6 (35.3)	0.016
Anti-NCS +, *n* (%)	14 (100.0)	12 (70.6)	0.027
Anti-APL +, *n* (%)	9 (64.3)	4 (23.5)	0.022
Hypocomplementemia, *n* (%)	12 (85.7)	9 (52.9)	0.052
SLEDAI-2 K, points	19.4 ± 6.0	7.5 ± 3.1	<0.001
Laboratory results			
WBC, ×10^9^/L	7.1 ± 3.0	6.6 ± 2.9	0.734
HGB, g/L	98.1 ± 19.3	104.2 ± 26.5	0.483
PLT, ×10^9^/L	209.9 ± 92.3	243.5 ± 101.8	0.369
ALB, g/L	34.0 ± 7.1	34.1 ± 9.6	0.984
BUN, mmol/L	8.7 ± 3.7	7.7 ± 3.3	0.720
Scr, umol/L	97.0 ± 32.8	72.2 ± 30.5	0.481
eGFR, ml/min	112.6 ± 41.5	131.2 ± 38.1	0.310
Hs-CRP, mg/L	4.5 ± 1.7	1.9 ± 0.6	0.113
PCT, ng/ml	0.23 ± 0.07	0.25 ± 0.08	0.475
PALB, mg/L	151.7 ± 52.9	203.5 ± 67.5	0.202
Total cholesterol, mmol/L	4.5 ± 1.3	4.7 ± 1.5	0.808
Triglycerides, mmol/L	2.1 ± 0.9	2.7 ± 1.1	0.384
HDL-C, mmol/L	1.1 ± 0.3	1.1 ± 0.5	0.767
LDL-C, mmol/L	2.5 ± 0.9	2.9 ± 1.1	0.651
LDH, U/L	331.6 ± 132.7	333.8 ± 133.6	0.964
Ca, mmol/L	2.1 ± 0.2	2.0 ± 0.1	0.120
C3, g/L	0.40 ± 0.21	0.77 ± 0.38	0.003
C4, g/L	0.08 ± 0.02	0.16 ± 0.07	<0.001

ZBA, *Z* score of body mass index for age; ZHA, *Z* scores of height for age; Anti-DNA, anti-double stranded DNA; anti-NCS, anti-nucleosome; APL, antiphospholipid; SLEDAI 2K, systemic lupus erythematosus disease activity index; WBC, white blood cells; HGB, hemoglobin; PLT, platelets; ALB, albumin; BUN, blood urea nitrogen; Scr, serum creatinine; eGFR, estimate glomerular filtration rate; hs-CRP, high-sensitivity C-reactive protein; PCT, procalcitonin; PALB, prealbumin; HDL-C, high-density lipoprotein cholesterol; LDL-C, low-density lipoprotein cholesterol; C3, complement component C3; C4, complement component C4.

**Figure 1:**
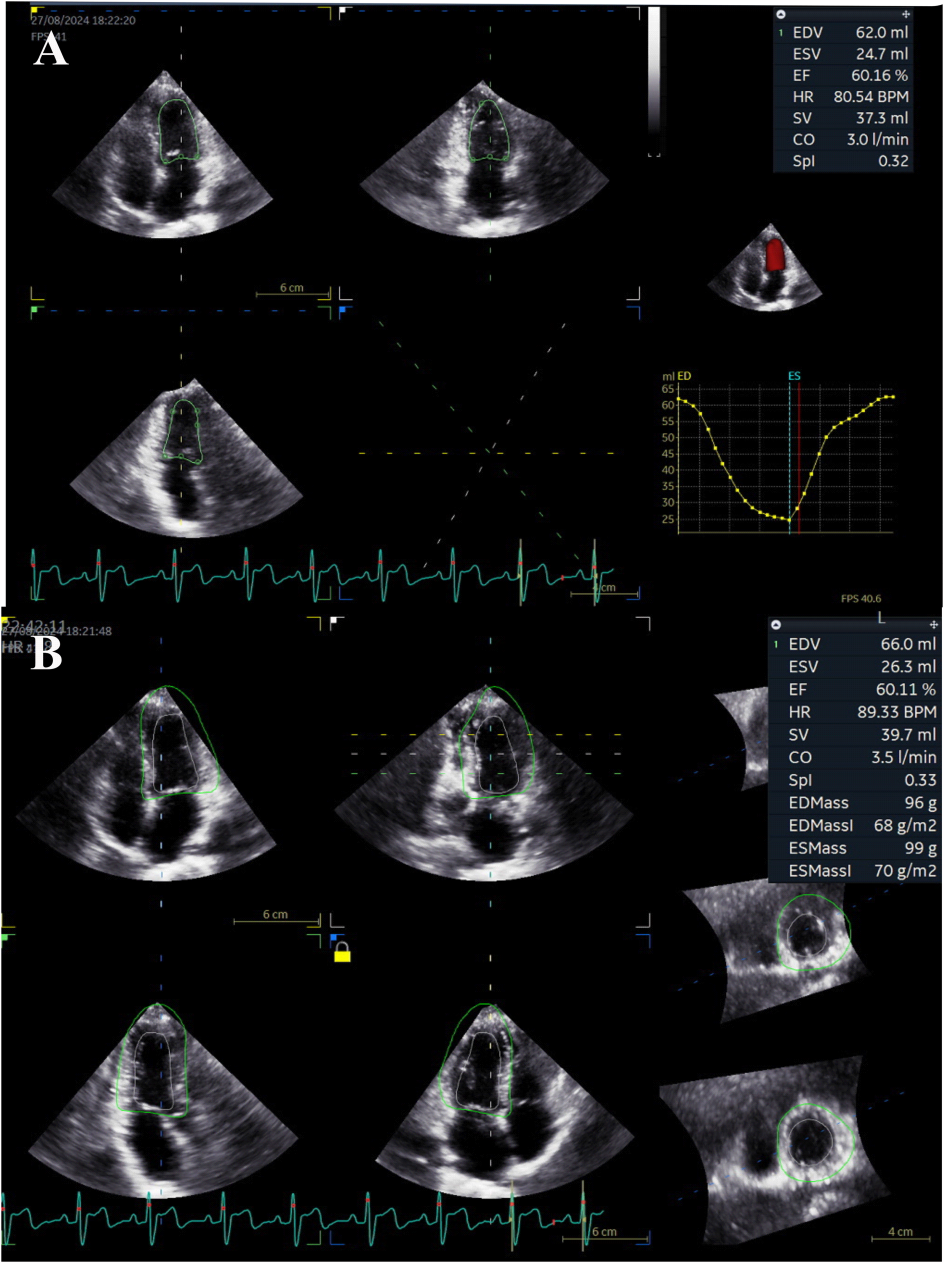
Demonstration of 3D-STI in a 14-year-old girl with SLE. **(A)** The 4D Automated Left Ventricular Quantification software presented the LV volume-time curve, end-diastolic volume (EDV), end-systolic volume (ESV), ejection fraction (EF), and stroke volume (SV) after identifying the track of endocardial border in a single cycle. **(B)** The 4D Automated Left Ventricular Quantification software traced the epicardial border and collected an entire delineation of the LV wall. EDV, end-diastolic volume; EF, ejection fraction; ESV, end-systolic volume; HR, heart rate; SPI, spherical index; SV, stroke volume.

**Figure 2:**
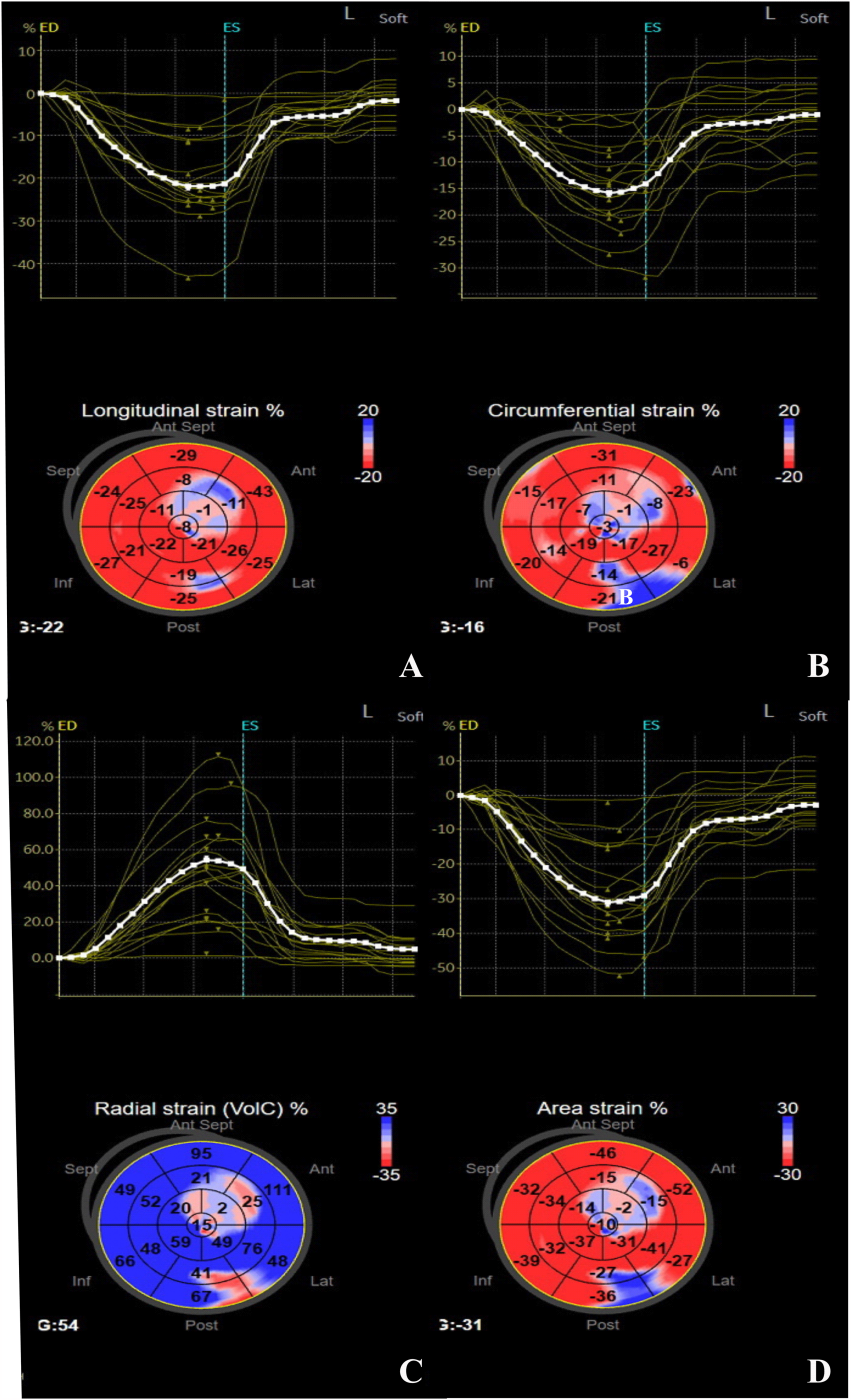
Strain-time curve and peak global systolic strain values were evaluated using the software in a 14-year-old girl with SLE. **(A)** GLS, **(B)** GCS, **(C)** GRS, **(D)** GAS.

Regarding the 3D-STI parameters, there was no statistically difference in LVEDV, LVESV, LVEF, SPI, and LV EDmass between the two groups (all *P* > 0.05). Simultaneously, there was a statistically significant rise in GLS, GCS, GAS, GRS, and LVtw in the mild-to-moderate group compared to the control group (all *P* < 0.05). No statistically significant difference was seen in Torsion and PSD between the mild-to-moderate group and the severe group (*P* > 0.05, Table 2, Figures 1, 2).

**Table 2:** Comparison of 3D conventional and strain parameters.

Characteristics	Severe group (*n* = 14)	Mild-to-moderate group (*n* = 17)	*P*-value
LVEDV, ml	80.0 ± 14.2	81.2 ± 12.6	0.808
LVESV, ml	36.9 ± 8.8	33.9 ± 8.0	0.330
LVEF, %	53.9 ± 7.7	58.3 ± 6.8	0.099
SPI, %	0.38 ± 0.06	0.40 ± 0.09	0.344
LV EDmass, g	92.4 ± 12.9	93.8 ± 13.7	0.772
GLS, %	16.0 ± 3.3	19.7 ± 3.6	0.006
GCS, %	13.4 ± 3.4	17.4 ± 4.0	0.006
GRS, %	40.1 ± 10.5	52.9 ± 14.4	<0.001
GAS, %	24.8 ± 5.3	32.0 ± 8.1	0.008
LVtw,°	3.4 ± 1.3	7.0 ± 3.1	0.019
Torsion,°/cm	0.86 ± 0.36	1.06 ± 0.46	0.253
PSD, ms	39.7 ± 12.3	36.5 ± 11.4	0.460

LVEDV, left ventricular end-diastolic volume; LVESV, left ventricular end systolic volume; LVEF, left ventricular ejection fraction; SPI, spherical index; LV EDmass, left ventricular end diastolic mass; GLS, global longitudinal strain; GCS, global circumferential strain; GRS, global radial strain; GAS, global area strain; LVtw, left ventricular twist angle; PSD, peak strain dispersion.

### The association between 3D-STI parameters and SLE disease activity

Univariate and multivariate logistic regression analyses were employed to assess the relationship between 3D-STI parameters and SLE disease activity. The findings indicated that GLS (odds ratio, OR, 0.69, 95% confidence interval, 95% CI, 0.50–0.96, *P* = 0.028) and LVtw (OR, 0.76, 95% CI 0.57–0.99, *P* = 0.047) were independently correlated with severe disease activity (Table 3).

**Table 3:** Logistic regression analysis of 3D-STI indicators for severe active of SLE patients.

Variables	Univariate			Multivariate		
	β	OR (95% CI)	*P*	β	OR (95% CI)	*P*
LVEDV	−0.007	0.99 (0.94–1.05)	0.801			
LVESV	0.045	1.05 (0.96–1.14)	0.321			
LVEF	−0.088	0.92 (0.82–1.02)	0.104			
SPI	−4.570	0.30 (0.10–108.33)	0.333			
LV EDmass	−0.009	0.99 (0.94–1.05)	0.763			
GLS	−0.326	0.72 (0.55–0.94)	0.017	−0.367	0.69 (0.50–0.96)	0.028
GCS	−0.314	0.73 (0.56–0.95)	0.020	0.467	1.60 (0.38–6.65)	0.522
GRS	−0.086	0.92 (0.85–0.98)	0.021	−0.203	0.82 (0.48–1.38)	0.451
GAS	−0.221	0.80 (0.67–0.96)	0.017	−0.308	0.74 (0.42–1.29)	0.283
LVtw	−0.235	0.79 (0.64–0.98)	0.032	−0.281	0.76 (0.57–0.99)	0.047
Torsion	−0.951	0.38 (0.07–2.01)	0.256			
PSD	0.024	1.03 (0.96–1.09)	0.447			
Constant				7.650		0.018

3D-STI, three-dimensional speckle tracking imaging; SLE, systemic lupus erythematosus; OR, odds ratio; 95% CI, 95% confidence interval; LVEDV, left ventricular end-diastolic volume; LVESV, left ventricular end systolic volume; LVEF, left ventricular ejection fraction; SPI, spherical index; LV EDmass, left ventricular end diastolic mass; GLS, global longitudinal strain; GCS, global circumferential strain; GRS, global radial strain; GAS, global area strain; LVtw, left ventricular twist angle; PSD, peak strain dispersion.

### Diagnostic consistency and performance of different diagnostic strategies

All diagnostic schemes were statistically effective in distinguishing the mild-to-moderate group from the severe group (all *P*-values <0.05). The diagnostic results shown reasonable consistency with SLEDAI findings for GLS (Kappa = 0.451) and LVtw (Kappa = 0.489), while the parallel scheme (Kappa = 0.618) and integrated scheme (Kappa = 0.672) demonstrated good agreement. Nonetheless, the serial technique was mostly congruent with SLEDAI results (Table 4).

**Table 4:** Consistency analysis between diagnostic patterns and SLEDAI results.

Index	Subtypes	SLEDAI result		Cohen's Kappa value (95% CI)	χ^2^	*P*-value
		Mild-to moderate	Severe			
GLS	Mild-to moderate	7	12	0.451 (0.155, 0.747)	6.419	0.011
	Severe	10	2			
LVtw	Mild-to moderate	9	14	0.489 (0.252, 0.726)	8.89	0.003
	Severe	8	0			
Serial scheme	Mild-to moderate	12	14	0.312 (0.083, 0.541)	4.910	0.027
	Severe	5	0			
Parallel scheme	Mild-to moderate	4	12	0.618 (0.346, 0.890)	11.889	0.001
	Severe	13	2			
Integrated scheme	Mild-to moderate	15	3	0.672 (0.409, 0.935)^a^	14.072	<0.001
	Severe	2	11			

SLEDAI, systemic lupus erythematosus disease activity index; CI, confidence interval; GLS, global longitudinal strain; LVtw, left ventricular twist angle.

^a^ The value indicates the highest diagnostic performance in this metric.

**Figure 3:**
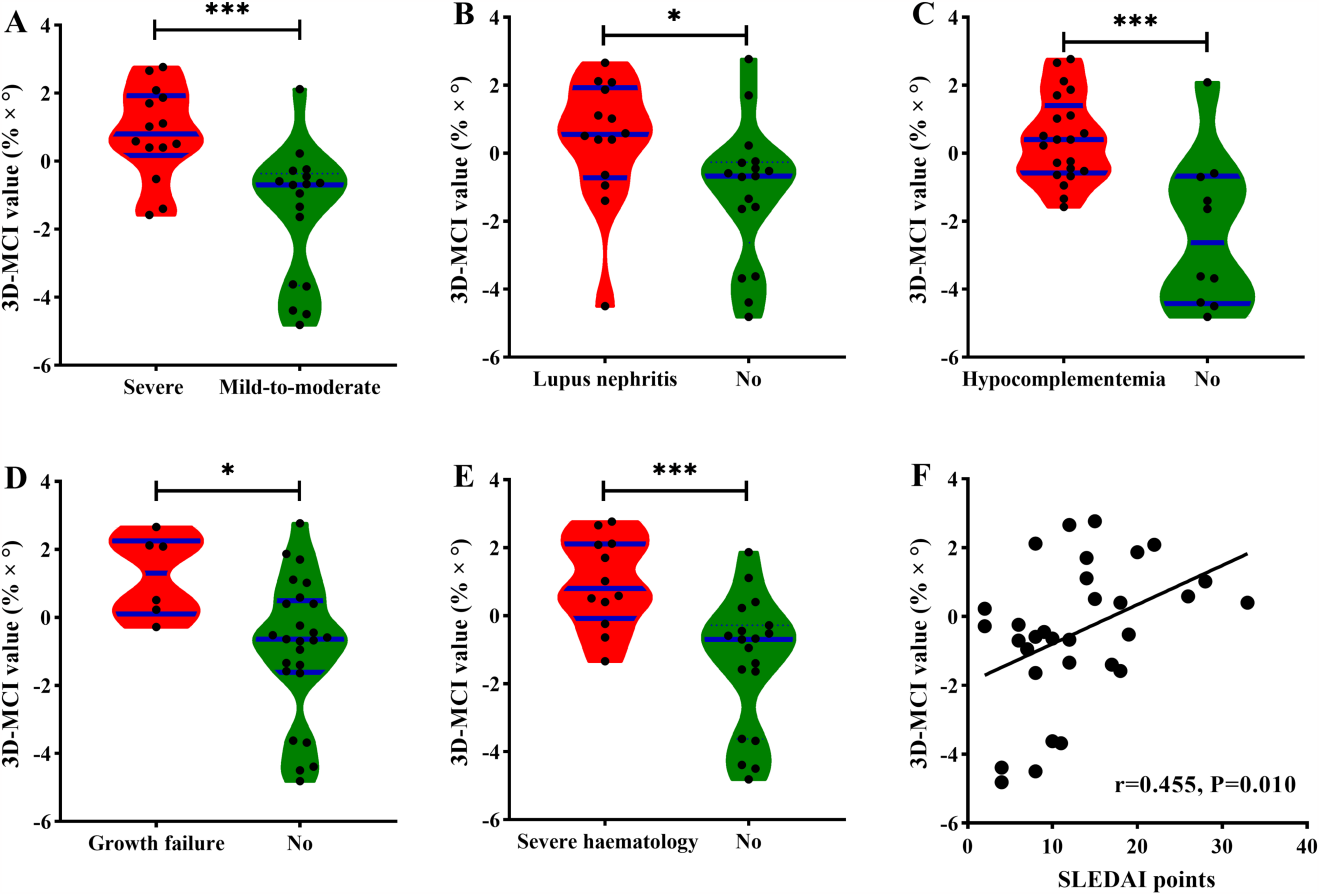
The distribution of 3D-MCI value in different clinical outcomes. **(A)** In the severe and mild-to-moderate group, **(B)** in the lupus nephritis and none group **(C)** in the hypocomplementemia and none group, **(D)** in the growth failure and none group, **(E)** in the severe hematology and none group, **(F)** the correlation between 3D-MCI value and SLEDAI points for all cSLE patients.

The best cutoff value was established based on the Youden index, with the GLS set at 18% and the LVtw value at 9.5° (Figure 2). The diagnostic formula, in alignment with the logistic regression technique, was incorporated into the integrated approach. 3D-MCI = 7.650–0.367*GLS - 0.281*LVtw. The Youden index determined the ideal threshold value of 3D-MCI for diagnostic probability to be 0.23. Table 5 and Figure 3A demonstrate that the integrated scheme for severe disease activity achieved commendable sensitivity and specificity, with values of 81.4% (95% CI 58.2–96.7%) and 94.1% (95% CI 71.3–99.9%), respectively. Furthermore, the AUC (85.8%; 95% CI: 80.0–91.6%), obtained from the integrated method, was quantitatively superior than all alternative techniques. Considering the diagnostic efficacy of the integrated strategy was notably extensive, the 3D-MCI was regarded as the best suitable diagnostic method for this medical condition.

**Table 5:** Comparison of diagnostic performance between various approaches.

Index	AUC	Sensitivity	Specificity	PPV	NPV
	95% CI	% (95% CI)	% (95% CI)	% (95% CI)	% (95% CI)
GLS	0.773 (0.588–0.903)	78.6 (49.2–95.3)	64.7 (38.3–85.8)	64.7 (47.7–78.7)	78.6 (55.9–91.4)
LVtw	0.685 (0.504–0.839)	85.7 (57.2–98.2)	58.8 (32.9–81.6)	63.2 (48.3–75.9)	83.3 (56.6–95.0)
Serial scheme	0.647 (0.506–0.809)	96.8 (76.8–100.0)	29.4 (10.3–56.0)	53.8 (46.2–61.3)	97.6 (76.2–100.0)
Parallel scheme	0.811 (0.630–0.928)	36.5 (20.1–63.2)	85.7 (57.2–93.2)	75.0 (55.4–87.9)	86.7 (63.7–96.0)
Integrated scheme	0.849 (0.675–0.951)	81.4 (58.2–96.7)	94.1 (71.3–99.9)	91.7 (61.7–98.7)	84.2 (66.0–92.9)

AUC, area under the curve; CI, confidence interval; PPV, positive predictive value; NPV, negative predictive value; GLS, global longitudinal strain; LVtw, left ventricular twist angle.

**Figure 4:**
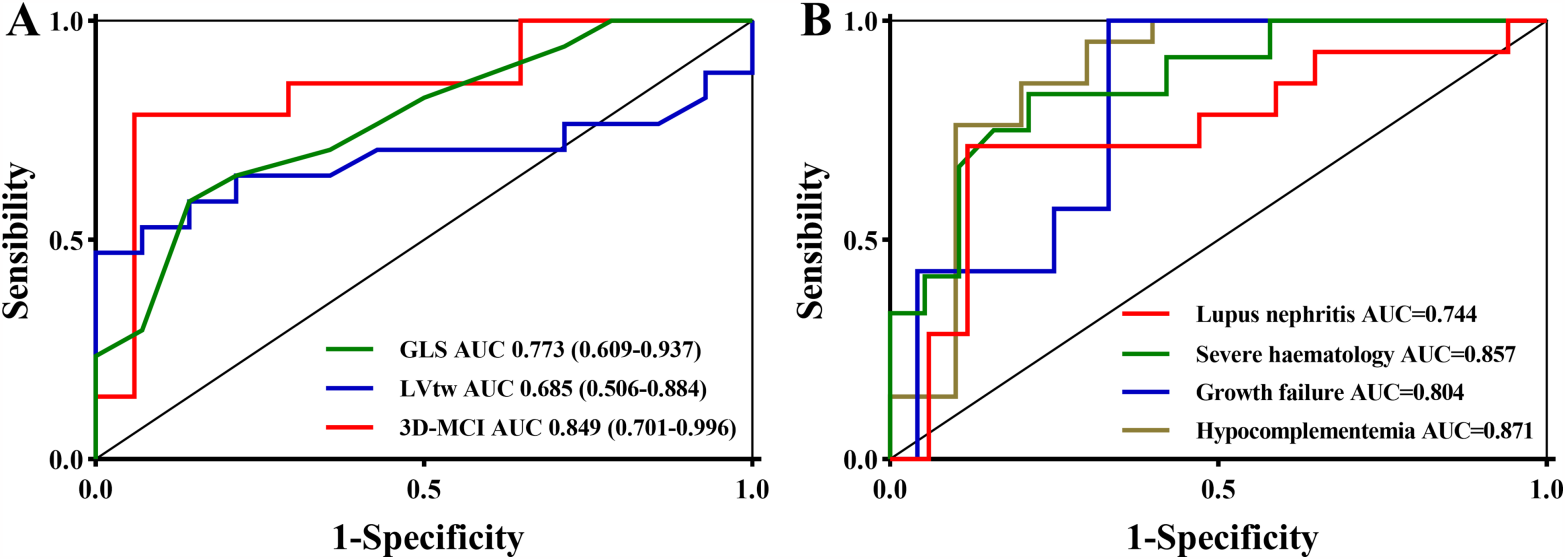
The receiver operator characteristic curve of GLS, lVtw, and 3D-MCI for the severe disease activity **(A)** and the receiver operator characteristic curve of 3D-MCI for the secondary outcomes **(B)**.

As for 3D-MCI value of the clinical outcomes, cSLE patients in the high 3D-MCI group had a higher level of SLEDAI points and lower levels of C3 and C4 (all *P* < 0.05). Patients with all incident secondary outcomes had a higher level of 3D-MCI value (Figures 4A–E). The 3D-MCI value was significantly correlated with SLEDAI points (Figure 4F). Moreover, high 3D-MCI group patients had higher proportion of severe disease activity, growth failure, lupus nephritis, hypocomplementemia, and serious hematological issues (Table 6 and Figure 3B).

**Table 6:** Clinical outcomes analysis of high and low 3D-MCI value for cSLE patients.

Outcomes	Low 3D-MCI value	High 3D-MCI value	Effect size	*P*-value
N	18	13	–	–
Primary outcome				
SLEDAI points	9.5 ± 2.8	17.5 ± 6.4	1.165	0.002
Severe disease activity	3 (16.7)	11 (84.6)	1.853	0.001
Secondary outcomes				
Growth failure	1 (5.6)	5 (38.6)	0.952	0.048
Lupus nephritis	4 (22.2)	10 (76.9)	1.307	0.008
Hypocomplementemia	9 (50.0)	12 (92.3)	1.056	0.036
C3, g/L	0.75 ± 0.28	0.40 ± 0.19	1.151	0.005
C4, g/L	0.15 ± 0.06	0.09 ± 0.04	0.843	0.031
Severe hematology	3 (16.7)	9 (69.2)	1.253	0.010
HGB, g/L	106.6 ± 26.5	94.3 ± 16.4	0.558	0.151
PLT, ×10^9^/L	253.5 ± 83.4	193.5 ± 66.2	0.600	0.107

SLEDAI, systemic lupus erythematosus disease activity index; C3, complement component C3; C4, complement component C4; HGB, hemoglobin; PLT, platelets.

### The association between 3D-MCI value for clinical outcomes

Table 7 presents the findings of the univariate and multivariable logistic regression analysis for 3D-MCI in a clinical context. The results indicated that the 3D-MCI value was significantly associated with severe disease activity in model 1 (OR, 2.72, 95% CI, 1.26–5.87, *P* = 0.011), model 2 (OR, 4.05, 95% CI, 1.42–11.55, *P* = 0.009), and model 3 (OR, 3.36, 95% CI, 1.17–9.69, *P* = 0.025) when treated as a continuous variable (Table 7). When the 3D-MCI was treated as a categorical variable, patients in the higher 3D-MCI group exhibited a significantly elevated risk of severe disease activity compared to those in the lower 3D-MCI group, after adjusting for three established models: model 1 (OR, 27.50, 95% CI, 3.91–193.49, *P* = 0.001), model 2 (OR, 28.95, 95% CI, 5.21–152.53, *P* = 0.004), and model 3 (OR, 40.33, 95% CI, 3.61–450.22, *P* = 0.003) (Table 7). Comparable outcomes were noted in the multivariate logistic regression study concerning 3D-MCI and lupus nephritis, hypocomplementemia, growth failure, and severe hematological conditions (Table 7). Moreover, The ROC analysis demonstrated that the 3D-MCI value could serve as good factor for different clinical outcomes (Figure 3B).

**Table 7:** The association between 3D-STI value and clinical outcomes.

Exposure	Model 1		Model 2		Model 3	
	OR (95% CI)	*P*	OR (95% CI)	*P*	OR (95% CI)	*P*
Severe activity						
3D-MCI as continuous	2.72 (1.26–5.87)	0.011	4.05 (1.42–11.55)	0.009	3.36 (1.17–9.69)	0.025
Low 3D-MCI	Ref.	–	Ref.	–	Ref.	–
High 3D-MCI	27.50 (3.91–193.49)	0.001	28.95 (5.21–152.53)	0.004	40.33 (3.61–450.22)	0.003
Lupus nephritis						
3D-MCI as continuous	1.56 (1.11–2.46)	0.009	1.80 (1.02–3.20)	0.046	2.03 (1.02–4.12)	0.045
Low 3D-MCI	Ref.	–	Ref.	–	Ref.	–
High 3D-MCI	11.67 (2.13–64.04)	0.005	22.34 (2.18–228.96)	0.009	21.61 (2.44–191.55)	0.006
Hypocomplementemia						
3D-MCI as continuous	2.71 (1.26–5.80)	0.010	2.44 (1.15–5.18)	0.020	2.66 (1.07–7.17)	0.043
Low 3D-MCI	Ref.	–	Ref.	–	Ref.	–
High 3D-MCI	15.06 (1.37–166.14)	0.027	12.01 (1.28–112.66)	0.030	8.28 (1.09–89.15)	0.041
Growth failure						
3D-MCI as continuous	2.00 (1.02–3.91)	0.044	2.58 (1.02–6.55)	0.046	2.77 (0.94–8.15)	0.064
Low 3D-MCI	Ref.	–	Ref.	–	Ref.	–
High 3D-MCI	5.00 (1.09–31.69)	0.048	6.85 (0.68–69.45)	0.103	5.80 (0.52–65.08)	0.154
Severe hematology						
3D-MCI as continuous	2.77 (1.27–6.03)	0.010	2.92 (1.26–6.79)	0.013	2.70 (1.12–6.48)	0.027
Low 3D-MCI	Ref.	–	Ref.	–	Ref.	–
High 3D-MCI	11.25 (2.04–62.20)	0.006	12.29 (1.83–82.29)	0.010	9.14 (1.25–67.10)	0.030

3D-MCI, three-dimensional myocardial comprehensive index; OR, odds ratio; 95% CI, 95% confidence index. Model 1 was unadjusted, model 2 adjusted for age, gender, body mass index. Model 3 adjusted for model 2 plus corticosteroid, hydroxychloroquine, anti-double stranded DNA, anti-nucleosome, antiphospholipid.

## Discussion

In addition to adult-onset SLE patients, cSLE patients may exhibit significant differences; the incidence of cSLE ranges from 0.36 to 2.5 per 100,000 children, with a prevalence of 1.89–34.1 per 100,000 (1, 14). In contrast to adult-onset SLE, cSLE exhibits a more aggressive course, characterized by elevated disease activity and a greater pharmacological burden, including corticosteroids and other immunosuppressive agents. This contributes to heightened morbidity and mortality, more severe organ manifestations, increased damage at diagnosis, and a higher prevalence of renal, cardiovascular, and neuropsychiatric complications. This study employed the 3D-STI approach to assess its clinical feasibility for cardiovascular lesions in individuals with cSLE (15, 16). We found that the 3D-STI serves as a comprehensive assessment tool for monitoring the initial phase of cardiovascular injury. Moreover, the 3D-MCI value, which enhanced the diagnostic efficiency of easily obtainable diagnostic variables through the development of several diagnostic techniques, may serve as an effective marker for disease activity and other issues associated with SLE. Collectively, our findings indicate that 3D-STI was a dependable technique for cSLE patients, and the suggested 3D-MCI value derived from LVtw and GLS shown a strong correlation with various SLE problems.

SLE is an autoimmune illness that can cause persistent damage to several organs, including the cardiovascular system, and is characterized by extended use of immunosuppressants and associated consequences. Cardiovascular impairment may represent one of the most severe consequences contributing to increased mortality (16). Nevertheless, the constraints of detection methodologies and the covert nature of early damage may result in the oversight of cardiovascular impairment in SLE patients, particularly in those with cSLE, thereby delaying subsequent preventive measures that might enhance their prognosis. Furthermore, transthoracic echocardiography is the primary modality for assessing cardiac impairment in SLE patients; yet, significant myocardial damage may occur, with a reduced likelihood of recovery, if cardiac injury is identified with two-dimensional ultrasound. This study revealed that some conventional ultrasonography measurements, including LVEDV, LVESV, and LVEF, remained within normal ranges in both the mild-to-moderate and severe groups. Consequently, there is an important need for doctors to have access to a non-invasive, user-friendly, quick, and somewhat accurate technology method for detecting cSLE and its associated problems.

In recent years, the swift advancement of detection technology, specifically the 3D-STI technique, transcends the constraints of two-dimensional ultrasound, facilitating a thorough analysis of left ventricular wall motion and accurately depicting the true condition of the left ventricle, thereby offering a novel perspective on cardiac structure and function. Prior research has shown that 3D-STI characteristics may provide a novel approach for the early and precise assessment of the mechanical properties and functional alterations of the cardiac system in cancer patients undergoing chemotherapy (9, 17, 18). A recent meta-analysis involving 1,515 breast cancer patients across 14 trials and revealed that 3D-STI is effective for the non-invasive and objective assessment of alterations in left ventricular function in breast cancer patients after anthracycline treatment (19). Few studies have identified a correlation between 3D-STI parameters and impaired LV deformation and dysfunction in individuals with intact LVEF across various illnesses (20, 21). Nonetheless, all of these investigations were undertaken with adult patients; to our knowledge, there has been just one previous research that only recruited juvenile patients. Kato et al. conducted observational research involving 52 cases of Kawasaki illness, with a median age of 2 years, and found no significant link between serum N-terminal pro-brain natriuretic peptide levels and global longitudinal strain on 3D speckle tracking imaging during the acute phase of Kawasaki disease (22). In contrast to this finding, our investigation revealed a significantly negative association between SLEDAI findings and GLS on 3D-STI (*r* = −0.368, *p* = 0.042). The 3D-MCI value, derived from GLS and LVtw, was significantly correlated with disease activity and several clinical sequelae in cSLE patients. In individuals with systemic lupus erythematosus (SLE), 3D-STI may be significant due to the premise that cardiac impairment is a primary early manifestation of SLE (23, 24). Dedeoglu et al. conducted a study utilizing 2D-STE on 35 patients with cSLE and 30 healthy children, revealing a significant decrease in the longitudinal strain of LV segments in cSLE patients. They concluded that 2D-STE could be beneficial in forecasting cardiovascular prognosis with novel therapeutic interventions (25). Furthermore, a recent study examined 30 adult SLE patients and 30 matched healthy individuals, revealing a statistically significant difference in 3D-STI parameters among the mild-to-moderate group, the severe group, and the healthy individuals. However, to our knowledge, no studies have investigated the relationship between 3D-STI and the prognosis of cSLE patients. This study demonstrated that 3D-STI is an effective approach for assessing cardiovascular impairment in cSLE patients. Moreover, the 3D-MCI value may function as an effective indicator of disease activity and other problems associated with SLE.

This study has some drawbacks. Initially, our patient group was limited in size and did not encompass healthy children. Secondly, several pertinent factors, such as the baseline SLEDAI score, were not included in this investigation; hence, this model was potentially overfitted. Thirdly, we did not do the 3D-STE at the disease's outset due to the method's unavailability at that time. Ultimately, treatment adherence was not evaluated in our cohort, and the study is constrained by its retrospective design and the restricted number of patients with specified outcomes, hindering more advanced statistical analysis.

## Conclusions

In summary, cSLE patients with normal LVEF may have subclinical myocardial damage, and 3D-STI can effectively monitor this early-stage myocardial subclinical injury in SLE. The novel measure, 3D-MCI, is especially pertinent for offering an objective imaging foundation for the early clinical identification of left ventricular myocardial involvement and for guiding prognosis in patients with cSLE.

## Data Availability

The raw data supporting the conclusions of this article will be made available by the authors, without undue reservation.
